# Effects of suspension of anti-vascular endothelial growth factor treatment for neovascular age-related macular degeneration in clinical setting

**DOI:** 10.1007/s00417-021-05526-0

**Published:** 2022-01-30

**Authors:** Hisashi Matsubara, Yoshitsugu Matsui, Ryohei Miyata, Atsushi Ichio, Shinichiro Chujo, Hiroko Enomoto, Masahiko Sugimoto, Mineo Kondo

**Affiliations:** 1 Department of Ophthalmology, Mie University Graduate School of Medicine, 2-174 Edobashi, Tsu City, Mie 514-8507 Japan; 2 Subarukai Eye Center, Higashiomi, Japan

**Keywords:** Age-related macular degeneration, AMD, Anti-VEGF agents, Intravitreal injection, Treatment suspension, Recurrence, Number of hospital visits

## Abstract

**Purpose:**

To investigate the outcomes of a suspension of anti-vascular endothelial growth factor (anti-VEGF) treatments in the eyes with neovascular age-related macular degeneration (nAMD).

**Methods:**

This was a retrospective study that examined eyes having no exudation for 48 weeks while undergoing intravitreal anti-VEGF injections every 12 to 16 weeks. The rate and time of recurrences, best-corrected visual acuity (BCVA), central subfield thickness (CST), number of visits, and reactivity to anti-VEGF were determined after the suspension of the anti-VEGF treatments.

**Results:**

In 34 eyes of 34 patients, 17 eyes (50.0%) had a recurrence during the 24-month follow-up period. The median time of a recurrence was 10 months. The BCVA was maintained for 24 months after the suspension regardless of the development of any recurrences. In 41.7% of the eyes that resumed treatment, the duration of exudation suppression by the anti-VEGF therapy was shorter than 12 weeks during the 12 months after restarting the anti-VEGF treatments. There was a significant increase in the number of visits during the first year after beginning the suspension versus during the 1 year before the suspension (non-recurrence group; *P* = 0.007, recurrence group; *P* = 0.001).

**Conclusion:**

Although one-half of the eyes had a recurrence within 24 months after a suspension of anti-VEGF treatment, the BCVA was maintained after a resumption of the anti-VEGF treatments. However, the number of hospital visits increases regardless of the recurrences and the lesion stability is altered by the anti-VEGF suspension. Clinicians should explain both the advantages and disadvantages of anti-VEGF suspension to nAMD patients.


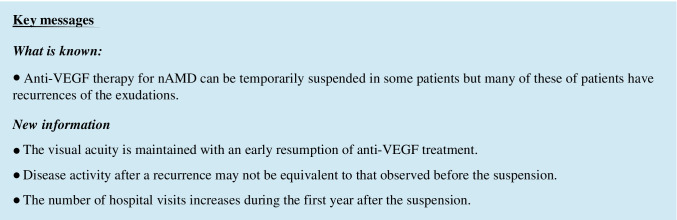


## Introduction

Age-related macular degeneration (AMD) is a major cause of vision reduction in the elderly, and it is feared that the number of cases will increase worldwide in the near future [1]. In Japan, there has been an increase in the number of patients with neovascular AMD (nAMD) in conjunction with the increase in the elderly population and westernization of their lifestyle [2, 3]. Today, intravitreal injections of anti-vascular endothelial growth factor (VEGF) agents have become the first-line therapy for nAMD. Several early nAMD clinical trials have shown that monthly or bimonthly injections of anti-VEGF agents were effective in improving and maintaining the visual acuity [4–6]. However, the need of monthly or bimonthly intravitreal injections imposed a heavy economic and physical burden on the individuals. On the other hand, treat and extend (TAE) regimen, in which the treatment interval is changed depending on the status of the retinal lesions, can reduce the burden on patients by decreasing the cost, number of injections, and hospital visits, with an improvement in the visual acuity [7, 8]. Moreover, to reduce the burden of the intravitreal injections, guidelines for the suspension of the anti-VEGF injections in eyes with nAMD with milder lesion activity have been proposed [9, 10]. In contrast, there is another proposal that these treatments should be continued to maintain the effects of the anti-VEGF treatments [11]. Thus, at present, it remains unclear as to whether suspension of anti-VEGF is effective in reducing the burden while maintaining the efficacy of the treatments. Thus, the purpose of this study was to evaluate the rate of recurrences and the degree of exudations in eyes with nAMD which matched the anti-VEGF suspension criteria, regardless of any past intravitreal injection treatment history. This study also examined the visual and anatomical outcomes and the changes in the number of hospital visits after the suspension of the anti-VEGF treatments.

## Materials and methods

### Study design

The procedures used were approved by the Ethics Committee of Mie University Hospital (approval number. H2020-021), and they conformed to the tenets of the Declaration of Helsinki. The medical records of nAMD patients who were treatment naïve and had started the treatment of the nAMD by intravitreal injections of ranibizumab (Lucentis; Novartis, Bulach, Switzerland) or aflibercept (Eylea; Bayer, Basel, Switzerland) between April 1, 2009, and December 31, 2018, at the Mie University Hospital, Mie, Japan, were reviewed.

### Inclusion and exclusion criteria

Patients were included if they were (1) > 50 years of age, (2) had been diagnosed and treated by intravitreal injections of ranibizumab or aflibercept for more than 2 years at the Mie University Hospital, (3) subsequently treated by the TAE regimen or the fixed dosing regimen, (4) agreed to the suggestion of treatment suspension, and (5) met the “suspension criteria.” The “suspension criteria” was defined as the absence of exudations or hemorrhages for at least 48 weeks with continuous anti-VEGF treatment every 12 to 16 weeks. Patients were excluded if they had photodynamic therapy, laser photocoagulation in the macular region, prior vitrectomy or intravitreal injection of steroids in the targeted eye, or had been treated in both eyes by an anti-VEGF agent during the study period for any retinal disease.

### Data acquisition

The beginning of the treatment suspension was defined as the visit when the last anti-VEGF injection was given during the “suspension criteria” period (baseline). The recurrence point was defined as the visit when subretinal fluid (SRF), intraretinal fluid (IRF), or retinal hemorrhage was detected by optical coherence tomography (OCT) or fundus examinations. The demographic and clinical features extracted from the medical records included the age, sex, past treatment periods (months), number of past anti-VEGF injections at the baseline, subtype of AMD, e.g., typical AMD or polypoidal choroidal vasculopathy (PCV), and the greatest linear dimension (GLD) of the lesion at the time of first diagnosis. The decimal best-corrected visual acuity (BCVA) and central subfield thickness (CST) were measured in all eyes at each visit. The pretreatment subtype of AMD was determined from the fluorescein and indocyanine green angiographic images. The CST was the average thickness in the central 1 mm, and it was measured as the distance between the internal limiting membrane and Bruch’s membrane in the OCT images.

### Post-suspension follow-up methods and treatment regimens

After the suspension of anti-VEGF treatments, the status of the lesion was monitored by OCT, fundus examinations, and BCVA measurements at each visit. The first visit after the suspension was at 3 months, and the subsequent visits were to our hospital or nearby clinic was every 1 to 2 months during the first year after the suspension. During the second year, the visit interval was decided on by the treating ophthalmologist, and the patients were examined every 2 to 4 months. This interval was continued until a recurrence occurred. In all eyes in which the anti-VEGF treatment was resumed, the presence of exudation was assessed at 1 month after the resumption. The treatment regimen used after the resumption was selected by the treating ophthalmologist.

### Number of hospital visits

The number of visits to the Mie University Hospital for the purpose of evaluation and treatment for the nAMD was counted during the following periods: (1) 1 year before the baseline (Pre 1 year), from the closest visit 12 months prior to the baseline to baseline, (2) the first year after suspension (Year 1), from the next visit after the baseline to the closest visit 12 months after the baseline, and (3) the second year after suspension (Year 2), from the next visit after the last visit in Year 1 to the closest visit 24 months after the baseline.

### Statistical analyses

The descriptive data are presented as numbers, percentages, medians with the first and third quartiles (Q1, Q3), and the 95% confidence intervals (CIs) when appropriate. The rate and recurrence times were analyzed using the Kaplan–Meier method. Cox proportional hazard regression analysis was performed to estimate the hazard ratio for the recurrences by univariate and multivariate analysis and included the age, sex, subtype of AMD, and past treatment period prior to the suspension. The selection of priori variables was based on the data in past publications [12, 13], the result of univariate analyses, and clinical perspectives. For statistical analyses, Mann–Whitney’s U test, Fisher’s exact tests, the Wilcoxon matched pairs signed-rank tests, and the Friedman tests with a post-hoc Dunn test were used. The decimal BCVA was converted to the logarithm of the minimum angle of resolution (logMAR) units for statistical analyses. All *P* values were two sided and a *P* < 0.05 was taken to be statistically significant. A Cox proportional hazard regression analysis was performed using the IBM SPSS Statistics Desktop (version 26; IBM Japan, Tokyo, Japan). Other statistical analyses were performed using Prism 8 software (GraphPad, Inc., La Jolla, CA, USA).

## Results

### Baseline characteristics

**Fig. 1:**
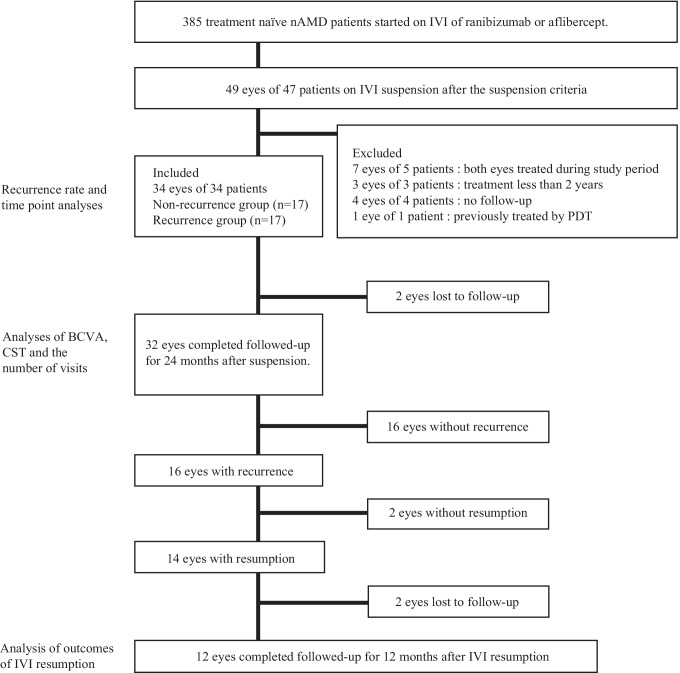
Flow chart showing the course of the patients. nAMD neovascular age-related macular degeneration; BCVA best-corrected visual acuity; CST central subfield thickness; PDT photodynamic therapy; CNV choroidal neovascularization; IVI intravitreal injection

Of the 385 patients whose medical records were examined, there were 34 eyes of 34 patients who met the inclusion criteria (Fig. 1). These included 12 females and 22 males whose median age was 76 years. The baseline characteristics of the patients are presented in Table 1. Of the 34 eyes, there were 21 eyes with typical AMD and 13 eyes with PCV. The median of the past treatment period was 39 months, and the median number of the past treatments was 14. A recurrence was found in 17 (50.0%) of the 34 eyes. The type of fluid in recurrent cases was SRF in 9 cases, IRF in 2 cases, both SRF and IRF in 4 cases, and retinal hemorrhage in 2 cases. Only the past injection numbers were significantly different between the non-recurrence and recurrence groups (*P* = 0.0451; Table 1).

**Table 1:** Baseline characteristics of patients with and without recurrence

		Overall	Non-recurrence	Recurrence	*P* value
Number of eyes		34	17	17	
Sex (females/males)		12/22	8/9	4/13	0.282*
Age (years)	Median [Q1, Q3]	76 [72, 82]	75 [69, 81]	79 [74, 83]	0.115**
Treatment period (months)	Median [Q1, Q3]	39 [24, 58.75]	30 [24, 43]	46 [33, 71]	0.0679**
Past number of injections	Median [Q1, Q3]	14 [12, 21.75]	12 [12, 14]	19 [13, 25]	0.0451**
Subtype of AMD (tAMD/PCV)		21/13	11/6	10/7	> 0.999*
GLD (μm)	Median [Q1, Q3]	1845.5 [1303.0, 2677.8]	1542.0 [1116.0, 2143.0]	2097.0 [1327.0, 2847.0]	0.193**
Anti-VEGF drug (ranibizumab/aflibercept)		12/22	8/9	4/13	0.282*
Treatment interval (12/14/16 weeks)		27/1/6	13/1/3	14/0/3	0.595*

*Anti-VEGF drug* anti-VEGF drug used during the suspension criteria period, *treatment interval* treatment interval during the suspension criteria period, *Q1* first quartile (25th percentile); *Q3* third quartile (75th percentile), *BCVA* best-corrected visual acuity, *logMAR* logarithm of the minimum angle of resolution, *AMD* age-related macular degeneration, *GLD* greatest linear dimension, *tAMD* typical age-related macular degeneration, *PCV* polypoidal choroidal vasculopathy. *Fisher’s exact test, **Mann–Whitney U test

### Recurrence rates and time points

**Fig. 2:**
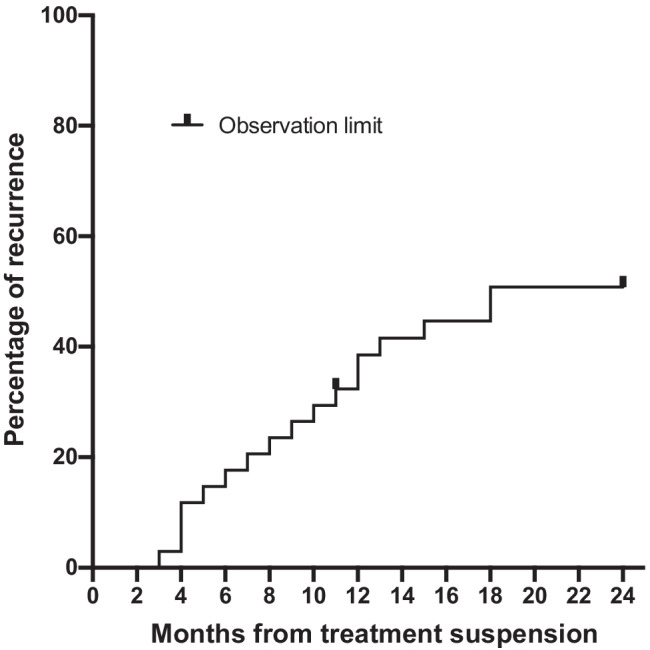
Kaplan–Meier survival plot showing the time and proportion of recurrences that occurred after the treatment suspension

By determining a recurrence by OCT at short intervals after the suspension, recurrences were detected on a monthly basis in most cases. Of the 34 eyes, 13 eyes (38.2%) had a recurrence within 12 months and 4 eyes (11.8%) had a recurrence within 13 to 24 months after the suspension of the anti-VEGF treatments (Fig. 2). Thus, 13 of the17 eyes (76.5%) had the recurrence occurring within 12 months. The median (Q1, Q3) time to recurrence after the treatment suspension was 10 (6, 12) months. The results of the Cox proportional hazard model are shown in Table 2. There were no factors that were significantly associated with the recurrence (*P* = 0.181).

**Table 2:** Cox proportional hazard model for all of the factors

	Hazard ratio	Lower 95% CI	Upper 95% CI	*P* value
Age	1.030	0.951	1.114	0.469
Sex	0.796	0.216	2.931	0.732
Subtype of AMD	1.502	0.528	4.271	0.445
Past treatment period	1.015	0.989	1.042	0.262

*AMD* age-related macular degeneration; *CI* confidential interval

### Changes of best-corrected visual acuity (BCVA) and central subfield thickness (CST)

The BCVA and CST for both subgroups at the baseline, at the time of a recurrence, and at 24 months after the suspension are shown in Table 3. The data for all 17 eyes with a recurrence are shown in Table 4. The BCVA did not change significantly in either subgroup (non-recurrence group, *P* = 0.424; recurrence group, *P* = 0.687). However, 2 eyes (cases #9 and #16) in the recurrence group had a worsening of the BCVA by > 0.20 logMAR units due to residual IRF in the macular lesion with and without treatment resumption. The CST in the recurrence group was significantly thicker at the time of the recurrence (*P* < 0.0001), and the thickness had recovered at 24 months after the suspension (*P* = 0.154).

**Table 3:** BCVA and CST of non-recurrence and recurrence groups at baseline, recurrence and 24 months after the suspension

		BCVA (logMAR)				CST (μm)					
		Baseline	Recurrence	24 months	*P* value	Baseline	Recurrence	24 months	*P* value	Baseline vs Recurrence	Baseline vs 24 months
Non-recurrence group (n = 16)	Median [Q1, Q3]	0.13 [0, 0.24]	-	0.22 [0, 0.33]	0.424*	251.0 [223.5, 292.3]	-	261.0 [234.0, 290.0]	0.197*	-	-
Recurrence group (n = 16)	Median [Q1, Q3]	0.19 [0, 0.22]	0.22 [0.16, 0.30]	0.22 [0.03, 0.30]	0.687**	247.0 [207.3, 270.8]	294.0 [279.0, 341.0]	281.5 [227.5, 306.5]	< 0.0001**	< 0.0001**	0.154**

*BCVA* best-corrected visual acuity, *logMAR* logarithm of the minimum angle of resolution, *CST* central subfield thickness

^*^Wilcoxon matched pairs signed-rank test, **Friedman test with post-hoc Dunn’s test multiple comparisons test

**Table 4:** Characteristics of patients with eyes exhibiting recurrence

No	Sex	Age	Subtype of AMD	Past treatment		Within the suspension criteria period		After the recurrence			For 12 months after the resumption		BCVA (logMAR)			
				Period (months)	IVI numbers	VEGF agent	IVI interval (weeks)	Recurrence (months)	IVI resumption	Treatment regimen	F/U	Complete suppression	Baseline	Recurrence	24 months after the suspension	12 months after the resumption
1	M	77	tAMD	78	28	A	12	3	Yes	FIX (12 weeks)	Yes	No	0.22	0.22	0.52	0.30
2	M	69	tAMD	33	16	A	12	4	Yes	FIX (12 weeks)	Yes	Yes	0.22	0.22	0.30	0.30
3	M	86	tAMD	92	37	A	12	4	Yes	FIX (12 weeks)	Yes	No	0.40	0.40	0.40	0.40
4	F	80	PCV	46	13	A	12	5	Yes	TAE (12 weeks ~)	Yes	No	0.10	0.22	0.046	0.046
5	M	79	PCV	71	25	A	12	6	Yes	PRN	Yes	Yes	0.00	0.00	0.00	0.097
6	M	64	PCV	70	21	A	12	6	Yes	FIX (12 weeks)	Yes	Yes	0.16	0.16	0.22	0.22
7	F	81	tAMD	24	12	R	12	7	Yes	TAE (12 weeks ~)	Yes	Yes	0.00	0.00	-0.079	0.00
8	M	74	PCV	49	16	A	12	9	Yes	FIX (12 weeks)	Yes	Yes	0.30	0.30	0.16	0.16
9	F	82	tAMD	24	12	R	12	10	No	-	No	-	0.82	1.3	1.7	-
10	M	72	PCV	40	13	A	16	11	Yes	TAE (16 weeks ~)	Yes	Yes	0.00	0.10	-0.079	-0.079
11	M	87	PCV	73	31	A	12	11	Yes	TAE (8 weeks ~)	No	-	0.22	0.30	-	-
12	M	83	PCV	42	22	A	16	12	Yes	TAE (12 weeks ~)	Yes	No	0.16	0.22	0.22	0.22
13	M	88	tAMD	38	19	A	12	12	No	-	No	-	0.22	0.52	0.30	-
14	F	66	tAMD	24	12	R	12	13	Yes	PRN	Yes	Yes	-0.079	-0.08	0.00	-0.079
15	M	91	tAMD	97	35	A	12	15	Yes	PRN	No	-	0.22	0.22	-0.30	-
16	M	79	tAMD	24	12	R	12	18	Yes	FIX (12 weeks)	Yes	No	-0.079	0.22	0.16	0.16
17	M	76	tAMD	55	19	A	16	19	Yes	TAE (8 weeks ~)	No	-	0.22	0.16	0.22	-

*Complete suppression* complete suppression of exudation for 12 weeks

*AMD* age-related macular degeneration, *tAMD* typical age-related macular degeneration, *PCV* polypoidal choroidal vasculopathy, *IVI* intravitreal injection, *VEGF* vascular endothelial growth factor, *F/U* follow-up, *BCVA* best-corrected visual acuity, *logMAR* logarithm of the minimum angle of resolution, *F* female, *M* male, *A* aflibercept, *R* ranibizumab, *TAE* treat and extend, *PRN* pro re nata

### Response to anti-VEGF agents after resumption

Of the 12 eyes that completed the 12 months of follow-up after the resumption of anti-VEGF treatments, 6 eyes were treated with a 12-week fixed regimen, 3 eyes had the TAE regimen starting at 12-week intervals, 1 eye underwent TAE starting at 16-week intervals, and 2 eyes underwent the pro re nata (PRN) regimen. The exudation disappeared in all of the resumed cases at 1 month after the resumption. However, in 5 of 12 (41.7%) eyes, the exudation suppression was not sustained for at least one 12-week period during the 12 months after the resumption.

### Number of visits before and after treatment suspension

**Fig. 3:**
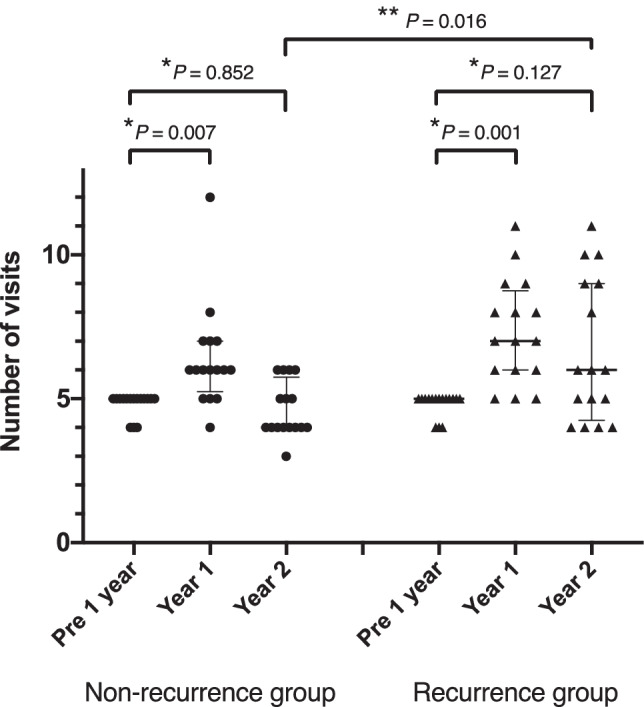
Dot plot showing the number of visits for the non-recurrence and recurrence groups before and after suspension of intravitreal anti-VEGF injections. Each dot represents an individual eye. Horizontal bars represent the median with the interquartile range. *Friedman test with post-hoc Dunn multiple comparison test. **Mann–Whitney U test

The number of visits before and after the suspension is shown in Fig. 3. In both subgroups, the number of visits was significantly increased between the Pre 1 year and Year 1 (non-recurrence group, *P* = 0.007; recurrence group, *P* = 0.001), but there were no significant differences between Pre 1 year and Year 2 (non-recurrence group, *P* = 0.852; recurrence group, *P* = 0.127). In contrast, the number of visits during Year 2 in the recurrence group was significantly greater than that for the non-recurrence group (*P* = 0.016). The 6 eyes in the recurrence group with 8 or more visits during Year 2 had recurrences at 7, 12, 13, 15, 18, and 19 months after the suspension.

### Number of injections that was reduced due to suspension of anti-VEGF treatments

The number of injections that was reduced by the suspension was calculated using the dosing interval during the “suspension criteria” period and the recurrence time points. The median (Q1, Q3) number of injections that were reduced for all eyes was 5 (3, 8), for the non-recurrence group was 8 (6, 8), and it was 3 (2, 4) for the recurrence group.

## Discussion

Our study was designed for real-world clinical conditions, and the findings showed that the rate of recurrences of exudation was 38.2% for 12 months and 50.0% for 24 months after the treatment suspension. There were no statistically significant factors that could be used to predict the time of the recurrence. In contrast, the BCVA was maintained for 24 months after the immediate resumption of the anti-VEGF treatment in the recurrence cases. We also gained a new insight that there was a significant increase in the number of hospital visits during the first year after the suspension regardless of whether an exudation recurred.

A suspension of the anti-VEGF treatment in eyes with nAMD has been proposed as part of the treatment protocol [9, 10] or the TAE regimen [12, 14–16]. Earlier studies reported that the rate of recurrence was 13.0 to 52.9% after an interruption of anti-VEGF treatment under the TAE regimen [12, 14–16]. In our study, we found a similar high recurrence rate after suspension of the intravitreal anti-VEGF treatments. These results indicated that it is necessary to monitor the treatment-suspended eyes with a careful search for recurrences because a stabilization of choroidal neovascularization (CNV) is not sustained in many cases. Moreover, several other studies have reported that the majority of recurrences occurred during the first year after the suspension [12–14] with a percentage as high as 50% [13, 14]. Our study also found that 76.5% of recurrences occurred during the first year after the suspension. Another result of this study indicated that recurrences occurred at an almost constant rate during the first year after the suspension. However, it should be noted that follow-up examinations were performed every 2 to 4 months in the earlier studies [12, 14, 15]. The eyes in our study were assessed for recurrence at 1- to 2-month intervals which is a closer interval compared to the earlier reports. Another study reported that the duration of VEGF suppression by anti-VEGF drugs varied from case to case [17]. Therefore, the discontinuation of the anti-VEGF drugs may have led to a recurrence at a time when the VEGF suppression was lost in these cases. As a result, variations in the timing of the recurrence could have caused this constant rate of recurrences which could be detected by our detailed evaluations. These results are similar to that of an earlier prospective study that used bimonthly follow-up examinations [12]. These findings along with those of the previous studies suggested that continuous and close follow-ups are required for at least 1 year after the suspension of the anti-VEGF treatments.

The BCVA was maintained for 24 months after the suspension regardless of recurrences which is similar to previous reports [14–16]. It is well known that the improvement of the BCVA in cases where the timing of the intravitreal injection of anti-VEGF agent is delayed can be difficult when exudation was present [18]. Therefore, the early detection of recurrences and the appropriate timing of the anti-VEGF injections are important factors in being able to maintain the BCVA. In the current study, it was possible to detect a recurrence earlier by our shorter intervals of evaluations. Based on these results, early therapeutic resumption of anti-VEGF treatment might be an effective way to maintain the BCVA in recurrence cases. However, it is similar to an earlier report that pointed out that the visual acuity reduction can occur in some cases regardless of the resumption of treatment [14]. For example, there was one eye that had a worsening of the visual acuity due to residual IRF in the macular region despite the resumption of the anti-VEGF treatment. The reasons for this may be associated with the change in the response to the anti-VEGF agent or inadequate treatment after the resumption. In our study, the short-term anti-VEGF response to the lesion was good and remained unchanged due to the absence of exudation at 1 month after the resumption in all resumed eyes. In contrast, the long-term drug responsiveness could have been altered because in 5 of 12 eyes (41.7%), the suppression of the exudation was not completely sustained for 12 weeks during the 12 months after the resumption of intravitreal anti-VEGF injections. These results suggest that the duration of the exudation inhibition was alterable under the anti-VEGF suspended conditions as compared to that observed before the suspension. Earlier studies have reported that the lesion activity was constant after 2 to 3 years of starting anti-VEGF treatments [13, 19], and the treatment interval was not changed during a long follow-up period when under continuous treatment [20]. All 5 eyes in this study were treated for more than 2 years prior to the suspension of anti-VEGF treatments. However, VEGF was not suppressed by the anti-VEGF suspension. As a result, the persistent lack of VEGF suppression might have led to an increase in the disease activity. Although the treatment regimen after resumption will likely affect the subsequent outcomes, the optimal retreatment regimen has yet to be definitively determined. In earlier studies, the TAE regimen was restarted after 2 or 3 monthly anti-VEGF injections [14, 15]. However, the eyes evaluated in our study were treated with various regimens after resuming the anti-VEGF treatments. Thus, the visual acuity of recurrence eyes had not deteriorated at the time of the recurrence and in all recurrence eyes exudation had completely disappeared at 1 month after the resumption of the treatments. These results may have led the treating doctors to judge that the disease activity was as low as at the time of suspension and that monthly dosing was not necessary. In 5 eyes that had unsatisfactory exudation control, 3 eyes were retreated with a 12-week fixed regimen while 2 underwent TAE restarting at the 12-week intervals. Thus, there is a possibility that the treatment during the early stages after the recurrence was inadequate. Therefore, to reduce and control the exudation quickly, it may be better to restart treatments with several consecutive monthly doses followed by the TAE regimen.

Although the number of visits represents one of the burden factors, there has been no study that has evaluated the number before and after the suspension of anti-VEGF treatments. Monthly evaluations are desirable after the suspension to detect recurrences earlier because the treatment regimen is changed from the proactive regimen to the PRN regimen. Therefore, the eyes evaluated in this study were assessed for recurrences at the shortest possible interval. As a result, the number of visits could have been significantly increased during Year 1 as compared to that at Pre 1 year regardless of recurrences. In contrast, during Year 2, the number of visits could have decreased because the follow-up interval in the non-recurrence eyes was extended because there were no recurrences for 1 year and also because the treatment was resumed in the recurrence eyes and continued with a long interval regimen in most of the eyes with a recurrence. However, in Year 2, there were a larger number of visits for the recurrence group than the non-recurrence group due to the recurrence cases that occurred and resumed during Year 2. Based on these results, the preferred follow-up period after the suspension would be a monthly evaluation during the first year, with the second year visit interval based on the first year results. The rationale for this method is to detect recurrence early so that treatment can be resumed even if more visits are required.

All of the results demonstrated the necessity of considering the advantages and disadvantages of suspending the anti-VEGF treatments. The advantages of suspension are the reduction in the treatment cost and the risk of complications that can occur from repeated intravitreal injections. Our results showed that it was possible to avoid a median of 8 anti-VEGF injections in our non-recurrence group over 2 years. In contrast, the disadvantages included the possibilities of visual acuity reduction, requirement of shorter dosing intervals after the resumption, and an increase of the number of hospital visits. Moreover, it has been reported that 79% of cases were reactivated within 5 years after a treatment suspension [13]. Therefore, recurrences are likely to occur over a long period of time in most cases, and thus, there is a limit to the number of intravitreal injection treatments that can be reduced. Appropriate considerations of each case are required as the advantages and disadvantages might be different for each case.

## Limitations

The limitations of this study include the small sample size and the retrospective design. In addition, this study included cases in which the treatment regimens were changed to proactive regimens due to higher levels of lesion activity even if initiated with the PRN regimen. Therefore, it is possible that the CNV lesion activity might not have been lower. In addition, the study design had a selection bias as patients who agreed to the suggestion of treatment suspension were included in the analyses. Therefore, the enrolled participants in this study were not selected purely on the basis of disease activity. There was also a limitation with regard to the standardization of the treatment regimen before and after suspension of the anti-VEGF treatments because this was a retrospective study. As a result, our overall outcomes might not be representative. Despite the above limitations, the results may be helpful in making decisions for intravitreal injection suspension in the real-world clinical practice.

## Conclusions

In conclusion, the findings demonstrate that one-half of the eyes that met the “suspension criteria” had a recurrence within 2 years. Thus, treatment suspension had both good and bad outcomes. Therefore, clinicians need to fully explain both possibilities to patients when anti-VEGF treatment suspension is being considered. Further research is required to assess the long-term outcomes of the anti-VEGF suspension in patients with nAMD in the real-world clinical setting.
